# Serum progranulin levels are elevated in dermatomyositis patients with acute interstitial lung disease, predicting prognosis

**DOI:** 10.1186/s13075-015-0547-z

**Published:** 2015-02-10

**Authors:** Atsushi Tanaka, Hiroshi Tsukamoto, Hiroki Mitoma, Chikako Kiyohara, Naoyasu Ueda, Masahiro Ayano, Shun-ichiro Ohta, Yasutaka Kimoto, Mitsuteru Akahoshi, Yojiro Arinobu, Hiroaki Niiro, Yoshifumi Tada, Takahiko Horiuchi, Koichi Akashi

**Affiliations:** Department of Medicine and Biosystemic Science, Kyushu University Graduate School of Medical Sciences, 3-1-1 Maidashi, Higashi-ku, Fukuoka 812-8582 Japan; Department of Preventive Medicine, Kyushu University Graduate School of Medical Sciences, Fukuoka, 812-8582 Japan; Department of Internal Medicine, Kyushu University Beppu Hospital, Oita, 874-0838 Japan; Department of Rheumatology, Faculty of Medicine, Saga University, Saga, 849-8501 Japan

## Abstract

**Introduction:**

Progranulin (PGRN), a pleiotropic growth factor, has emerged as an immunoregulatory molecule. Because the roles of PGRN in dermatomyositis (DM) are still unknown, we investigated whether serum PGRN levels are associated with disease activity and prognosis in DM patients, particularly in those with DM complicated with interstitial lung disease (ILD).

**Methods:**

The serum levels of PGRN were measured by enzyme-linked immunosorbent assay in patients with DM (n =57; acute/subacute interstitial pneumonia (A/SIP): n =17, chronic interstitial pneumonia (CIP): n =24, without ILD: n =16), polymyositis (PM, n =21; including 6 with ILD) and normal healthy controls (NHCs, n =60). We assessed the correlation between the serum PGRN levels and the activity indexes of ILD or prognosis in DM patients with ILD.

**Results:**

Serum PGRN levels were significantly higher in DM patients than in PM patients (*P* =0.0025) and in NHCs (*P* <0.0001). In DM patients, the levels were significantly higher in patients with A/SIP than in those with CIP (*P* <0.0001) or without ILD (*P* =0.0003). The serum PGRN levels in DM patients with ILD significantly correlated with serum ferritin (*r*_S_ =0.77, *P* <0.0001), lactate dehydrogenase (*r*_S_ =0.54, *P* =0.0003) and C-reactive protein (*r*_S_ =0.48, *P* =0.0015) levels. Moreover, in DM patients with ILD, the cumulative survival rate for 6 months was significantly lower in the group with serum PGRN levels ≥200 ng/ml (67%) than in the group with serum PGRN levels <200 ng/ml (100%) (*P* =0.0009).

**Conclusions:**

Serum PGRN is associated with disease activity and prognosis of DM with ILD. PGRN may play a role in the pathogenesis of DM and could be a useful biomarker.

## Introduction

Dermatomyositis (DM) is a systemic autoimmune and inflammatory disease that involves not only the muscle and skin but also several other organs, such as the lungs, heart and joints [1]. Interstitial lung disease (ILD) is the most common internal organ manifestation, and it affects the prognosis of DM patients. ILD is classified into two subsets: acute/subacute interstitial pneumonia (A/SIP) and chronic interstitial pneumonia (CIP) [2,3]. A/SIP is often complicated with clinically amyopathic DM (CADM) [4,5], which shows the typical skin manifestations of DM but has no or little evidence of clinical myositis [6]. A/SIP complicated with CADM is life-threatening and shows a rapidly progressive pattern, with a 6-month survival rate of 40.8%, irrespective of intensive therapy [5]. Combination therapy with prednisolone (PSL), cyclosporine A (CsA) and intravenous pulse cyclophosphamide (IVCY) is more effective for A/SIP with DM than PSL plus one immunosuppressive agent [7]. Therefore, diagnosis and evaluation of ILD is very important to determine the treatment strategy when DM is diagnosed [8-10]. Although several biomarkers that may reflect inflammatory activity of lung have been utilized, such as ferritin [11-14] and Krebs von den Lungen-6 (KL-6) [15-19], there are no established serum biomarkers for DM-associated ILD.

Progranulin (PGRN) [GenBank:NC_000017] is an extracellular glycoprotein containing seven and one-half repeats of cysteine-rich motifs. PGRN is proteolytically cleaved by extracellular proteases, such as proteinase 3 (PR3) and elastase, into granulin (GRN) [20] that range from 6 to 25 kDa. PGRN is abundantly expressed in rapidly cycling epithelial cells, leukocytes, chondrocytes and neurons [21], and its expression level is at steady state [22]. PGRN plays a critical role in early embryogenesis [22], wound healing [23], maintenance of neuronal survival [24] and tumorigenesis [21]. Particularly, macrophage-derived PGRN is a key regulatory factor in the processes of inflammation and wound healing [23,25]. Recent mouse studies show that mice unable to convert PGRN into GRN due to lack of both elastase and PR3 cannot show inflammation in response to injection of immune complexes [26]. These data indicate that PGRN is synthesized by macrophages and is cleaved into GRNs by elastase in tissues to enhance inflammation. Moreover, PGRN and/or GRN act as a soluble cofactor for Toll-like receptor 9 (TLR9) signaling and enhance it [27]. We previously reported that serum PGRN levels are significantly elevated in systemic lupus erythematosus (SLE) patients in parallel with disease activities and that PGRN may have a role in the pathogenesis of SLE, partly by enhancing the TLR9 signaling and interleukin (IL)-6 production [28]. Although macrophage activation is considered to underlie the pathogenesis of A/SIP with DM [11-14] and TLR9 and IL-6 is associated with DM [29-35], the roles of PGRN in DM are still unknown.

Here, we show that serum PGRN levels are significantly elevated in DM patients, particularly those with A/SIP, and are associated with disease activity and prognosis of DM patients with ILD. PGRN may have a role in the pathogenesis of DM.

## Methods

### Patients

We performed a cross-sectional study of patients who were treated for DM or polymyositis (PM) at Kyushu University Hospital, Saga University Hospital and Japanese Red Cross Fukuoka Hospital between 2002 and 2013. Fifty-seven Japanese patients with DM and twenty-one patients with PM were enrolled, and sera were obtained from these patients. All of the patients fulfilled the criteria of Bohan and Peter [36] for DM or PM or criteria for CADM [37]. We defined active DM and PM patients who required the initiation or reinforcement of treatment (overall disease activity). We excluded patients with other autoimmune and infectious diseases. Each patient completed a standardized medical history, including drug use, and was given a physical examination. The information obtained from the medical records of the patients included demographic data, such as age, sex, clinical manifestations of DM and laboratory values. Serological profiling of each patient, including white blood cells (WBCs), lymphocytes, hemoglobin (Hb), platelets (Plt), liver enzymes (aspartate aminotransferase (AST), alanine aminotransferase (ALT), γ-glutamyl transpeptidase (γ-GTP)), creatine kinase (CK), lactate dehydrogenase (LDH), ferritin, KL-6, C-reactive protein (CRP), anti-nuclear antibody (ANA) and anti-Jo-1 antibody, was performed using the standard methods. The serum samples from the patients with active DM were acquired before the initiation or reinforcement of treatment, and the samples from the patients with inactive DM were acquired during regular hospital visits. The samples were stored at −20°C. The active DM patients were treated with corticosteroids or immunosuppressive drugs after the completion of these evaluations. The sera obtained from five DM patients with ILD were reevaluated after ILD was ameliorated by treatment. Control sera were obtained from healthy staff members (n =60) at Kyushu University Hospital. This study was approved by the ethics committees of Kyushu University Hospital, Saga University Hospital and Japanese Red Cross Fukuoka Hospital, and the principles of the Declaration of Helsinki were followed throughout the study. Informed consent was obtained from all participants.

### Measurement of PGRN

The serum PGRN levels were determined using enzyme-linked immunosorbent assay (ELISA) kits (R&D Systems, Minneapolis, MN, USA) according to the manufacturer’s protocol. Briefly, calibrators, control sera and patients’ sera (stored samples) were diluted and incubated with a mouse monoclonal antibody against PGRN adsorbed onto the microtiter plate wells. After washing, a mouse monoclonal antibody against PGRN conjugated to horseradish peroxidase was added, followed by a second washing step and the addition of tetramethylbenzidine substrate. The intensity of the blue color developed was in proportion to the amount of PGRN bound in the initial step. The reaction was terminated by the addition of 2 N sulfuric acid. The absorbance was measured in a microtiter plate reader (Thermo Fisher Scientific, Waltham, MA, USA) and converted into nanograms per milliliter by plotting the values against the PGRN titer of the calibrators/standards given by the manufacturer. The assay range was 1.56 to 100 ng/ml.

### Classification of ILD

ILD was assessed by radiography and high-resolution computed tomography of the chest. ILD with DM was divided into two forms: A/SIP and CIP. A/SIP is defined as a rapidly progressive ILD within 3 months from the onset of symptoms and radiographically diffuse alveolar damage pattern or nonspecific interstitial pneumonias (NSIP). CIP is defined as an asymptomatic, non–rapidly progressive ILD or slowly progressive ILD over the course of 3 months and radiographically confirmed NSIP or bronchiolitis obliterans with organizing pneumonia pattern, according to the guidelines of the International Consensus Statement of idiopathic Pulmonary Fibrosis of the American Thoracic Society and the European Respiratory Society [38]. The designation of A/SIP and CIP was made blindly without knowledge of the laboratory results.

### Statistical analysis

The differences between two groups were analyzed using the Mann–Whitney *U* test. Fisher’s exact test was used for comparison of frequencies. The Steel-Dwass test was used for multiple comparisons among all groups. The relationships between PGRN levels and other continuous variables were analyzed using Spearman’s rank correlation. Receiver operating characteristic (ROC) analyses were performed for a mathematical expression of different serum PGRN levels as cutoff points. The cumulative survival rate was calculated using the Kaplan-Meier test. The log-rank test was also used to compare survival. *P*-values less than 0.05 were considered significant. All tests were two-tailed. All analyses were performed using JMP statistical software (SAS Institute, Cary, NC, USA).

## Results

### Serum PGRN levels were markedly elevated in patients with DM

Of the 57 patients with DM enrolled in the present study, 49 were women, and 8 were men (active: n =40, inactive: n =17). These patients ranged in age from 30 to 87 years (median age: 53 years). Of the 21 patients with PM, 16 were women, and 5 were men (active: n =11, inactive: n =10). These patients ranged in age from 15 to 85 years (median age: 53 years). Among the NHCs, 51 were women, and 9 were men. They ranged in age from 20 to 59 years (median age: 32 years). There were significant differences between patients with DM or PM and NHCs in terms of age. However, there was not a significant correlation between serum PGRN levels and age in NHCs.

**Figure 1:**
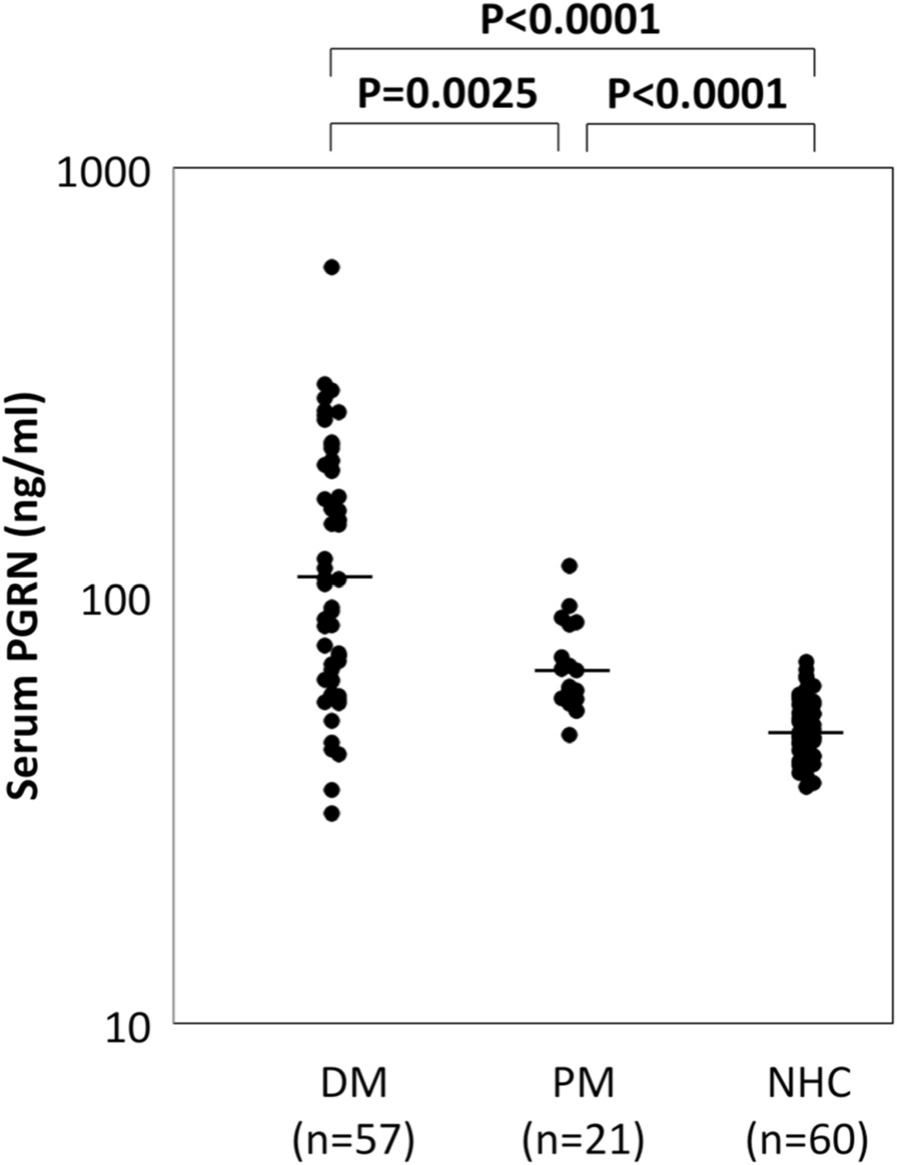
**Serum progranulin levels were elevated in patients with dermatomyositis.** Serum progranulin (PGRN) levels in 57 patients with dermatomyositis (DM), 21patients with polymyositis (PM) and 60 normal healthy controls (NHCs), as measured by enzyme-linked immunosorbent assay. The serum PGRN levels in DM patients were significantly higher than those in NHCs.

To investigate the association of PGRN with DM, we first compared serum PGRN levels among 57 patients with DM, 21 patients with PM and 60 NHCs by using ELISA (Figure 1). Serum PGRN levels in NHCs were always within the range of 35 to 70 ng/ml and distributed normally. Serum PGRN levels in patients with PM (median: 60 ng/ml) were significantly higher than in healthy controls (48 ng/ml) (*P* <0.0001). Strikingly, serum PGRN levels in patients with DM (median: 109 ng/ml) were significantly and markedly higher than in PM patients (*P* =0.0025) and in healthy controls (*P* <0.0001).

### Serum PGRN levels were elevated in DM patients with A/SIP

**Figure 2:**
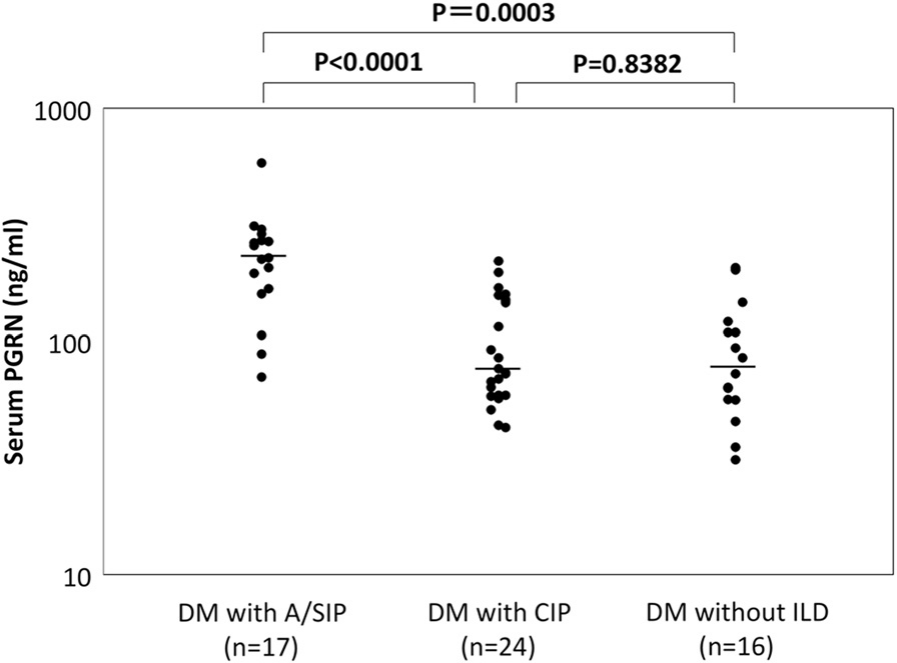
**Serum progranulin levels were elevated in dermatomyositis patients with acute/subacute interstitial pneumonia.** Serum progranulin (PGRN) levels in 17 patients with dermatomyositis (DM) with acute/subacute interstitial pneumonia (A/SIP), 17 with chronic interstitial pneumonia (CIP) and 16 without interstitial lung disease (ILD) are shown, as measured by enzyme-linked immunosorbent assay. The serum PGRN levels in patients with DM with A/SIP were significantly higher than in DM patients without A/SIP.

Because DM is occasionally complicated with ILD [8-10], we next compared serum PGRN levels among DM patients with A/SIP (n =17), those with CIP (n =24) and those without ILD (n =16) (Figure 2). Serum PGRN levels in DM patients with A/SIP (median: 228 ng/ml) were significantly higher than in DM patients with CIP (75 ng/ml, *P* <0.0001) or without ILD (79 ng/ml, *P* =0.0003). There was no significant difference between serum PGRN levels in DM patients with CIP and those without ILD (*P* =0.8382). However, serum PGRN levels in DM patients with CIP or without ILD were significantly higher than in NHCs (*P* <0.0001 and *P* =0.005, respectively; data not shown).

### Comparison of clinical manifestations between DM patients with A/SIP and without A/SIP

To evaluate clinical manifestations other than serum PGRN levels, we compared those between DM patients with A/SIP and without A/SIP (Table 1). There were no significant differences among all the subsets in terms of age and sex. The duration between the onset and evaluation in DM patients with A/SIP was significantly shorter than in those without A/SIP. The frequency of muscle weakness was significantly lower in DM patients with A/SIP than in those without it, indicating that the ratio of CADM patients was significantly higher in DM patients with A/SIP than in those without it. WBCs and Hb tended to be lower in DM patients with A/SIP than in those without it. Although CK levels showed no significant difference among all the subsets, AST and ALT levels were significantly higher in DM patients with A/SIP than in those with CIP and without ILD. The levels of LDH, ferritin, KL-6 and CRP, which are used as serologic parameters for disease activity of ILD, showed significant difference between DM patients with A/SIP and those without A/SIP. The frequency of ANA or anti-Jo-1 positivity was not significantly different among all the subsets.

**Table 1:** **Comparison of clinical manifestations between dermatomyositis patients with acute/subacute interstitial pneumonia and without acute/subacute interstitial pneumonia** ^a^

**Variables**	**DM with A/SIP**	**DM with CIP**	**DM without ILD**
Number of patients	17	24	16
Age, yr	54.0 (36 to 65)	54.5 (19 to 78)	36 (15 to 85)
Female, n (%)	14 (82.4)	20 (83.3)	15 (93.8)
Duration, mo	2 (1 to 19)	33 (1 to 148)^***^	11 (1 to 237)^**^
Muscle weakness, n (%)	4 (23.5)	15 (62.5)^*^	15 (94.1)^***^
WBCs, count/ml	5,200 (2,900 to 12,300)	8,165 (2,900 to 14,990)	7,590 (3,540 to 11,580)
Hb, g/dl	11.6 (7.9 to 13.9)	12.8 (10 to 15.4)	12.5 (9.8 to 15.3)
Plt, ×10^5^/μl	22.2 (14.9 to 34.5)	23.0 (12 to 38.8)	20.6 (6.2 to 37.7)
CK, IU/L	209 (33 to 866)	1,289 (16 to 12,300)	568 (51 to 2,646)
AST, IU/L	74 (31 to 219)	30 (14 to 657)^**^	44 (12 to 132)^*^
ALT, IU/L	42 (19 to 111)	20 (11 to 301)^*^	31 (10 to 150)
γ-GTP, IU/L	43 (16 to 346)	24 (10 to 131)	26 (10 to 165)
LDH, IU/L	392 (225 to 957)	273 (186 to 1,975)^*^	328 (157 to 489)^*^
Ferritin, ng/ml	875 (251 to 2,171)	88 (16 to 4,853)^***^	105 (39 to 400)^***^
KL-6, U/ml	959 (338 to 2,048)	525 (188 to 1,802)^*^	224 (125 to 459)^***^
PGRN, ng/ml	228 (70 to 586)	75 (43 to 221)^***^	79 (31 to 207)^***^
CRP, mg/dl	0.86 (0.03 to 2.97)	0.24 (0.03 to 3.66)^**^	0.08 (0.01 to 0.9)^***^
ANA >160, n (%)	1 (5.9)	2 (8.3)	5 (31.3)
Anti-Jo-1, n (%)	0 (0)	4 (16.7)	0 (0)

^a^ANA, Anti-nuclear antibody; A/SIP, Acute/subacute interstitial pneumonia; ALT, Alanine aminotransferase; AST, Aspartate aminotransferase; CIP, Chronic interstitial pneumonia; CK, Creatine kinase; CRP, C-reactive protein; DM, Dermatomyositis; γ-GTP, γ-glutamyl transpeptidase; Hb, Hemoglobin; ILD, Interstitial lung disease; KL-6, Krebs von den Lungen-6; LDH, Lactate dehydrogenase; Plt, Platelets; WBC, White blood cell. The values of age, duration, CK, AST, ALT, γ-GTP, LDH, ferritin, KL-6, PGRN and CRP indicate median (range). *P <0.05, **P <0.01, ***P <0.001 compared with DM with A/SIP using the Mann–Whitney *U* test or Fisher’s exact test.

### Serum PGRN levels in DM patients with ILD correlated with disease activities of ILD

**Figure 3:**
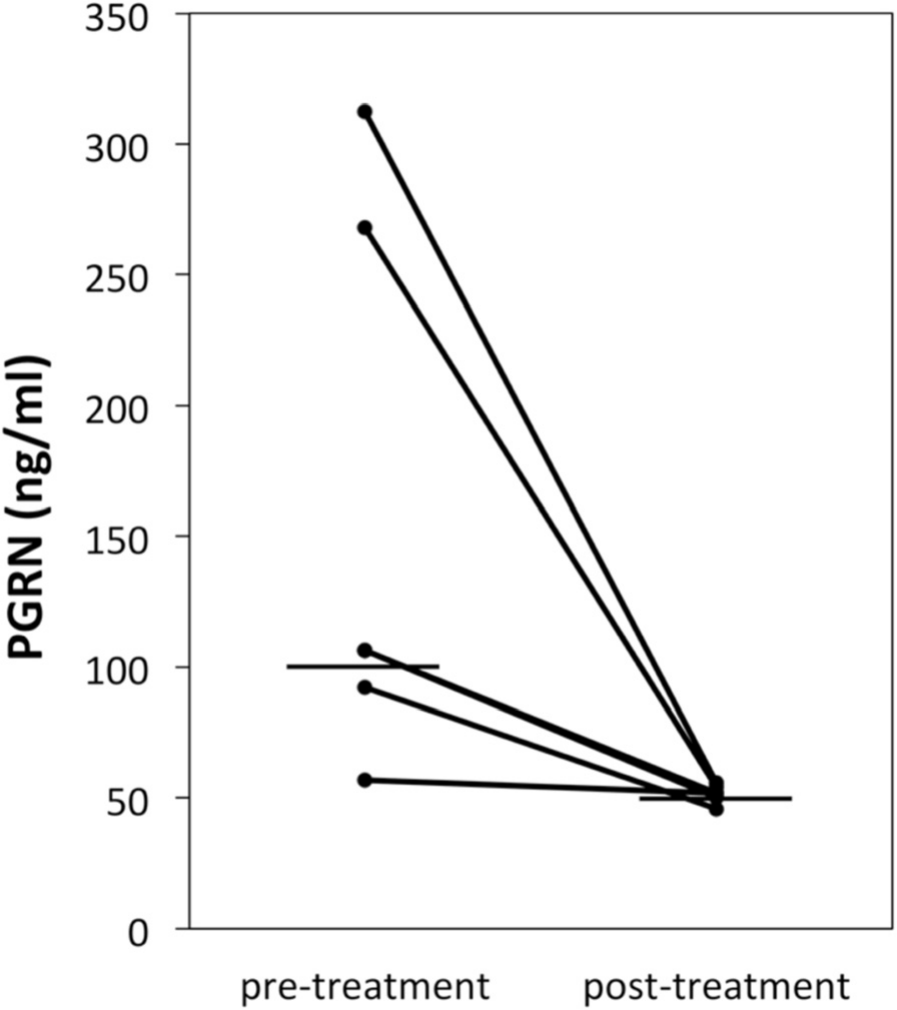
**Serum progranulin levels are decreased after ameliorating the interstitial lung disease with treatment.** Serum progranulin (PGRN) levels before and after treatment in five patients with active interstitial lung disease. The serum PGRN levels decreased with the clinical amelioration of the disease.

We next tested whether serum PGRN levels in DM patients with ILD correlate with conventional serologic parameters for disease activity of ILD (Table 2). Serum PGRN levels showed a significantly positive correlation with serum levels of LDH (*r*_S_ =0.54, *P* =0.0003), ferritin (*r*_S_ =0.77, *P* <0.0001) and CRP (*r*_S_ =0.48, *P* =0.0015). However, there was not a significant correlation between serum levels of PGRN and KL-6 (*r*_S_ =0.26, *P* =0.1034). In five DM patients with active ILD that had subsided with treatment, serum PGRN levels decreased in all of them (Figure 3). These results indicate that serum PGRN levels reflect disease activity of ILD in DM patients.

**Table 2:** **Serum progranulin levels in dermatomyositis patients with interstitial lung disease correlate with disease activity of interstitial lung disease** ^a^

**Variables**	***r***_S_	***P*** **-value**
PGRN vs LDH	0.54	0.0003
PGRN vs ferritin	0.77	<0.0001
PGRN vs KL-6	0.26	0.1034
PGRN vs CRP	0.48	0.0015

^a^CRP, C-reactive protein; KL-6, Krebs von den Lungen-6; LDH, Lactate dehydrogenase; PGRN, Progranulin; *r*_S_, Correlation coefficient established employing Spearman’s correlation coefficients. Correlations between the serum PGRN levels and LDH, ferritin, KL-6 and CRP are shown, as measured in 41 DM patients with ILD.

### Treatment

The frequency of treatment with PSL alone was fairly low in patients with DM with A/SIP, whereas the frequency of treatment with immunosuppressive agents in addition to PSL was significantly higher in DM patients with A/SIP than in DM patients without ILD (*P* =0.0004) (Table 3). Immunosuppressive agents used in patients with DM with A/SIP were calcineurin inhibitors (CNIs: CsA or tacrolimus) for nine patients and the combination therapy with CNI plus IVCY for three patients. We performed hematopoietic stem cell transplants (HSCTs) for two refractory DM patients with A/SIP, irrespective of the combination therapy with PSL, CNI plus IVCY, and could rescue them [39].

**Table 3:** **Treatment in each group** ^a^

**Treatments**	**DM with A/SIP**	**DM with CIP**	**DM without ILD**
Number of patients	17	24	16
PSL or other agent alone, n (%)	3 (18)	8 (33)	12 (75)**
PSL	3	7	11
CNI	0	1	0
AZA	0	0	1
PSL + other agents, n (%)	14 (82)	16 (67)	3 (19)**
CNI	9	13	1
MTX	0	0	2
IVCY	0	1	0
CNI + MZB	0	1	0
CNI + IVCY	3	0	0
HSCT	2	1	0

^a^A/SIP, Acute/subacute interstitial pneumonia; AZA, Azathioprine; CIP, Chronic interstitial pneumonia; CNI, Calcineurin inhibitor (cyclosporine or tacrolimus); DM, Dermatomyositis; HSCT, Hematopoietic stem cell transplant; ILD, Interstitial lung disease; IVCY, Intravenous pulse cyclophosphamide; MTX, Methotrexate; MZB, Mizoribine; PSL, Prednisolone. **P <0.01 compared with DM with A/SIP using Fisher’s exact test.

### Serum PGRN levels in DM patients with ILD were associated with prognosis

**Figure 4:**
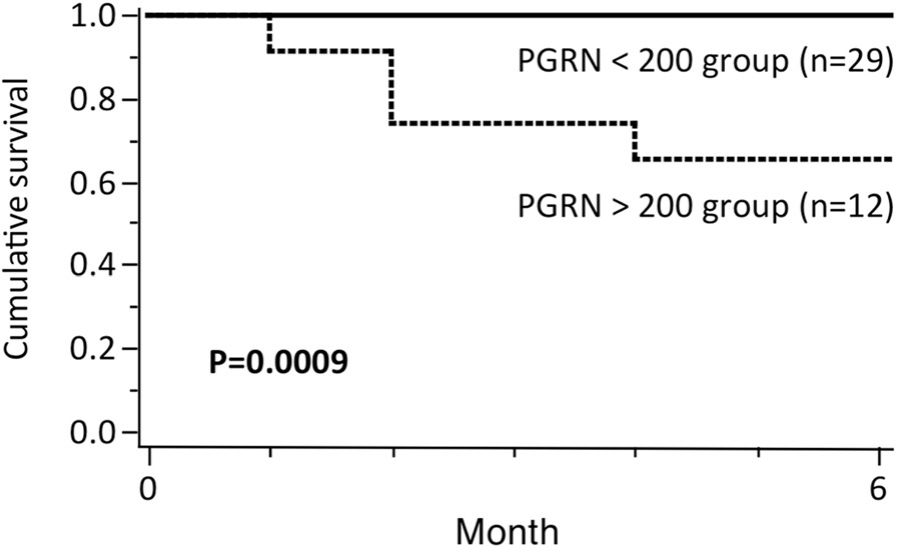
**Serum progranulin levels in dermatomyositis patients with interstitial lung disease were associated with prognosis.** The cumulative survival rate for 6 months in the serum progranulin (PGRN) <200 ng/ml group and the ≥200 ng/ml group of patients with dermatomyositis with interstitial lung disease are shown. The cumulative survival rate was calculated using the Kaplan-Meier test. The log-rank test was also used to compare survival. The cumulative survival rate was significantly lower in the group with serum PGRN levels ≥200 ng/ml than in the group with serum PGRN levels <200 ng/ml.

Because DM with ILD is occasionally life-threatening [4,5,7], in order to define the optimal cutoff point with the highest diagnostic accuracy in terms of survival, we performed ROC curve analysis for distinct serum PGRN levels. The highest area under the curve was calculated for a baseline serum PGRN level of 200 ng/ml. The two subsets were classified by serum PGRN level of 200 ng/ml. The cumulative survival rates for 6 months were 66.7% and 100% in the group with serum PGRN levels ≥200 ng/ml (n =12), including two patients treated with HSCT, and the group with serum PGRN levels <200 ng/ml (n =29) in DM with ILD, respectively (Figure 4). The cumulative survival rate was significantly lower in the group with serum PGRN levels ≥200 ng/ml than in the group with serum PGRN levels <200 ng/ml (*P* =0.0009). These results suggest that baseline serum PGRN levels predict the prognosis of DM patients with ILD.

## Discussion

To our knowledge, this is the first study to show that PGRN levels were significantly elevated in sera of DM patients, in particular those with A/SIP, and these concentrations were associated with the disease activity and prognosis of DM patients with ILD.

Consistent with previous reports [11,12], the elevation of transaminases and hyperferritinemia were observed in DM with A/SIP in the present study. In addition, WBC and Hb tended to be lower in DM patients with A/SIP than in those without it. These facts suggest that macrophages are substantially activated and cause injury to the lungs and the liver in DM patients with A/SIP. Actually, the accumulation of ferritin-producing macrophages has been shown in a CADM-related acute interstitial pneumonia autopsy case [40]. Because PGRN is highly expressed in macrophages [22], elevation of serum PGRN levels in DM with A/SIP also reflects macrophage activation as that of ferritin.

In the present study, other than DM, we also measured concentrations of serum PGRN in patients with PM (n =21), including 6 with ILD (median: 59 ng/ml) and 15 with systemic sclerosis (median: 49 ng/ml) (same levels in NHCs), including 13 with ILD (median: 49 ng/ml). In our data, although sera from PM patients contained significantly elevated PGRN compared with NHCs, DM patients’ sera had much higher levels of PGRN, especially among DM patients who also had A/SIP. This difference may occur because activated macrophages appeared especially in DM patients with A/SIP. Although we could not recruit acute respiratory distress syndrome (ARDS) patients into this study, ARDS could be an additional control to determine whether PGRN is a more general marker of acute respiratory inflammation.

PGRN is reported to be important in the initiation of inflammation by recruiting fibroblasts, macrophages and neutrophils to the site of inflammation [23]. PGRN is also converted to GRN by the elastase that is produced by leukocytes and other cells [41], however, and recent mouse studies have shown that GRN possesses inflammatory functions [25,26,42]. Thus, PGRN may be converted to GRN in the lungs and may be associated with the early phase of pathogenesis in DM with A/SIP. However, it is difficult to investigate the function of GRN in human studies, because we cannot measure GRNs at this moment.

Serum PGRN levels showed a significantly positive correlation with serum levels of LDH (*r*_S_ =0.54, *P* =0.0003) (Table 2) and ferritin (*r*_S_ =0.77, *P* <0.0001). However, there was no significant correlation between serum levels of PGRN and those of KL-6, another biomarker for ILD. Recent studies suggest serum ferritin levels as a marker for severity of acute progressive ILD in DM and CADM patients [11-14]. The serum ferritin concentrations in CADM patients with A/SIP are already elevated in early stages of the disease, before progression of ILD [11]. LDH and surfactant protein D are released into the blood when pulmonary cells are damaged with inflammation [43]. KL-6 rather reflects the regeneration and proliferation of pneumocytes after the damage of the pulmonary tissue [44]. Thus, PGRN could also be an acute marker for ILD as well as ferritin and LDH.

When we compared clinical manifestations between DM patients with A/SIP and without A/SIP, the frequency of CADM was significantly higher in those with A/SIP (76.5%) than in those with CIP (37.5%) and in those without ILD (5.9%). CADM may be a useful indicator for the occurrence of A/SIP. The levels of CRP showed significant differences between DM patients with A/SIP and those without and correlated with serum PGRN levels (*r*_S_ =0.48, *P* =0.0015) (Table 2). We previously reported that serum PGRN levels are elevated in SLE patients and that PGRN stimulated IL-6 production via TLR9 [28]. Upregulated IL-6 production stimulated by PGRN [27,28,45] may be related to elevation of CRP in DM patients with A/SIP. Serum IL-6 levels were significantly higher in dead than alive CADM patients with ILD [33] and were reported to be candidate biomarkers for disease activity in DM [32] and SLE [46]. Because TLR9 is highly expressed in DM [29-31] and SLE [47], upregulated IL-6 production stimulated by PGRN via TLR9 may be a common pathogenesis of DM and SLE. However, the proposed role of PGRN signaling via TLR9 in DM must be directly demonstrated in future studies. Elevated serum PGRN levels have been noted for a number of other pathological conditions [48]. So, PGRN is most likely not a disease-specific marker, but rather is probably a marker of immune activation.

Most of the DM patients with CIP were treated with PSL and immunosuppressive agents. HSCT was performed for one DM patient with CIP and a refractory skin ulcer. In contrast, several patients with A/SIP required combination therapy with PSL, CNI and IVCY (and HSCTs). This distinction might be responsible for cellular phenotypes affecting the pathogenesis of ILD.

The cumulative survival rate was significantly lower in the group with serum PGRN levels ≥200 ng/ml than in the group with serum PGRN levels <200 ng/ml (*P* =0.0009) (Figure 4). These data indicate that baseline serum PGRN levels can predict survival. The group with serum PGRN levels ≥200 ng/ml included two refractory patients with A/SIP treated with HSCTs, which saved their lives. If we had not been able to rescue them, the survival rate in the group with serum PGRN levels ≥200 ng/ml would have been lower. It is inferred from our findings that the intensive combination therapy with various immunosuppressive agents should be chosen for DM patients with ILD showing high serum PGRN levels, especially ≥200 ng/ml.

We must acknowledge some limitations of this study. The sample size of our study was small. Statistical tests usually require a larger sample size to justify that the effect did not happen by chance alone. Moreover, owing to the study’s cross-sectional design, it is difficult to establish the exact and definite causal relationships, except the association between PGRN and development of ILD with DM, on the basis of the collected data. There were potential confounding effects of medication use on serum PGRN levels in patients with inactive DM with CIP and without ILD. Last, to fully validate PGRN as a predictive biomarker, we need to show that elevated PGRN levels predate clinical respiratory decompensation.

## Conclusions

In conclusion, this pilot study demonstrates that the serum PGRN levels were elevated in patients with DM, in particular those with A/SIP, and were correlated with disease activity and prognosis of DM patients with ILD. PGRN could be a useful biomarker for disease activity and a predictor of survival in DM patients with ILD. These findings of the study will provide new insights into the pathogenesis as well as the therapy of DM, and will shed new light on the dysregulation of the immune system in autoimmune diseases. Further studies are required to reveal more precisely the mechanisms of PGRN in human autoimmune diseases.

## Abbreviations

A/SIP: Acute/subacute interstitial pneumonia
ALT: Alanine aminotransferase
ANA: Anti-nuclear antibody
ARDS: Acute respiratory distress syndrome
AST: Aspartate aminotransferase
AZA: Azathioprine
CADM: Clinically amyopathic dermatomyositis
CIP: Chronic interstitial pneumonia
CK: Creatine kinase
CNI: Calcineurin inhibitor
CRP: C-reactive protein
CsA: Cyclosporine A
DM: Dermatomyositis
ELISA: Enzyme-linked immunosorbent assay
γ-GTP: γ-glutamyl transpeptidase
GRN: Granulin
Hb: Hemoglobin
HSCT: Hematopoietic stem cell transplantation
IL: Interleukin
ILD: Interstitial lung disease
IVCY: Intravenous pulse cyclophosphamide
KL-6: Krebs von den Lungen-6
LDH: Lactate dehydrogenase
MTX: Methotrexate
MZB: Mizoribine
NHC: Normal healthy control
NSIP: Nonspecific interstitial pneumonia
PGRN: Progranulin
Plt: Platelet
PM: Polymyositis
PR3: Proteinase 3
PSL: Prednisolone
ROC: Receiver operating characteristic
SLE: Systemic lupus erythematosus
TLR: Toll-like receptor
WBC: White blood cell
